# *Brucella melitensis* VirB12 recombinant protein is a potential marker for serodiagnosis of human brucellosis

**DOI:** 10.1186/s12941-017-0182-4

**Published:** 2017-03-03

**Authors:** Shiva Mirkalantari, Amir-Hassan Zarnani, Mahboobeh Nazari, Gholam Reza Irajian, Nour Amirmozafari

**Affiliations:** 1 Microbiology Department, Faculty of Medicine, Iran University of Medical Sciences, Tehran, Iran; 2 Dept. of Immunology, School of Public Health, Tehran University of Medical Sciences, Tehran, Iran; 3 Immunology Research Center, Iran University of Medical Sciences, Tehran, Iran; 4 Monoclonal Antibody Reaserch Center, Avicenna Research Institute, ACECR, Tehran, Iran

**Keywords:** Brucellosis, VirB12, Recombinant proteins, Enzyme-linked immunosorbent assay, *Brucella melitensis*

## Abstract

**Background:**

The numerous drawbacks of current serological tests for diagnosis of brucellosis which mainly results from cross reactivity with LPS from other gram-negative bacteria have generated an increasing interest to find more specific non-LPS antigens. Previous studies had indicated that Brucella VirB12 protein, a cell surface protein and component of type IV secretion system, induces antibody response during animal infection. However, this protein has not yet been tested as a serological diagnostic marker in human brucellosis.

**Methods:**

Recombinant VirB12 protein was prepared and evaluated the efficacy of it in an indirect enzyme-linked immunosorbent assay (ELISA) for brucellosis with sera collected from different region of Iran and the results were compared with a commercial ELISA kit.

**Results:**

Sera from human brucellosis patients strongly reacted to the purified recombinant VirB12. The sensitivity, specificity, accuracy, negative predictive value and positive predictive value of recombinant VirB12-based ELISA related to the commercial-ELISA method were 87.8, 94, 90, 80 and 96.6% respectively.

**Conclusions:**

We concluded that antigenic VirB12 have a property value that can be considered as a candidate for using in serodiagnostic tests for human brucellosis.

## Background

*Brucella* spp. is a facultative intracellular pathogen that can be involved with many tissues and organs leading to a chronic infection, Brucellosis, in animals and humans [1–5]. Brucellosis is caused by several species of the genus Brucella including *Brucella abortus*, *Brucella melitensis*, *Brucella suis*, *Brucella canis*, *Brucella ovis*, and *Brucella neotomae* [6–9]. *B. melitensis* is the most frequently isolated species which is endemic in many developing countries [10, 11]. Clinical manifestations of brucellosis are very similar to other febrile diseases; therefore, the clinical diagnosis of this disease remains a challenge [12]. Common laboratory tests include either bacteriological culture of the pathogen or serological titration of anti-Brucella antibody. Although the gold standard test is said to be bacteriological isolation, the success rate of blood cultures is around 70–80% of cases in acute disease produced by *B. melitensis* [13]. This rate is very much lower in chronic cases. Various factors including disease duration, isolation method, and prior antibiotic intake can drastically impact the bacteriological diagnosis [2]. Serum agglutination serological tests often rely on detection of antibody against smooth lipoplysaccharide (LPS) present on bacterial cell surface. Due to existence of extensive cross reactivity with LPS from other gram negative bacteria, the specificity of these approaches are poorly suited for use in general diagnostic laboratories [2, 14–18]. The drawbacks of these classical serological tests have generated an increasing interest in finding more specific non-LPS based antigen candids [2, 19, 20]. In this regard, the outer membrane proteins of Brucella species have been proposed as appropriate candidate for antigenic component. Rolan et al. [21] noted that Brucella VirB12 protein, a component of type IV secretion system, which is situated on bacterial cell surface, is expressed during infection and induces an antibody response in cattle. However, they had not looked at any possible humoral response in humans during active or chronic infections. In a previous communicate we reported cloning of the virB12 gene of *B. melitensis* [22]. In the present study, we are reporting expression and purification of the recombinant VirB12 protein. Furthermore, the seroreactivity of the purified recombinant virB12 of *B. melitensis* was evaluated with human serum samples in an indirect enzyme-linked immunosorbent assay (ELISA) for brucellosis.

## Methods

### Preparation and recognition of Brucella VirB12 recombinant protein

The plasmid construct pET28a-VirB12 was purified from an overnight culture of *Escherichia coli* DH5α cell. The construct was transformed into competent *E. coli* Bl21 (ED3) cells. The cell harboring recombinant plasmid was spread on Luria-Bertani (LB) agar culture medium containing kanamycin. After verification, the transformed *E. coli* BL21 cells harboring the PET28a-VirB12 plasmid were used in the expression study. A single colony of the transformed cell was incubated overnight in 2 ml LB broth medium containing kanamycin (100 µl/ml) at 37 °C with constant shaking (200 rpm). The next day, 500 µl of culture materials was removed and incubated in 200 ml LB broth. The culture was grown to an OD_600_ nm of 0.6 with vigorous shaking (200 rpm) at 37 °C. Isopropyl-β-d- thiogalactopyranoside (IPTG) was added to a final concentration of 1 mM for expression of VirB12 recombinant protein. The incubated period was continued for another 4 h at 37 °C with shaking at 200 rpm. For analysis of production of the expressed protein, bacterial suspension were tested at 2 and 4 h intervals and analyzed on 12% SDS-PAGE. Following the fermentation process, cells were harvested by centrifugation at 6000×*g* for 15 min at 4 °C. Supernatants were discarded and cell pellets were frozen at −70 °C. The cell pellets were suspended with 10 mM Na_2_HPO_4_, 10 mM NaH_2_PO_4_ and 500 mM NaCl (pH, 7.4) and disrupted by sonication for 2 min. After sonication, the mixture was centrifuged at 15.200×*g* for 15 min at 4 °C. The pellet containing insoluble recombinant VirB12 protein (inclusion bodies) was washed three times with 50 mM Tris-Hcl, 10 mM EDTA, 100 mM NaCl and 0.5% Triton–X100 (pH 8). The pellet was resuspended in buffer containing 8 M urea, 10b mM TrisHcl (pH 7.4) and solubilized for 4 h by stirring at room temperature. The solubilized inclusion body was centrifuged at 15.200×*g* for 40 min at 4 °C, and the supernatant was collected. Affinity chromatography Ni-NTA column was used to purify VirB12. Were loaded onto Ni2—charged Hitrap column pre-equilibrated with 8 M urea in 20 mM sodium phosphate buffer, pH 7.4. VirB12 was eluted using a linear gradient with imidazol (10-500 mM) in 8 M urea, pH 7.4. Protein purification was monitored by 280 nm absorbance. Recombinant protein was analyzed by 12% sodium–dodecyl sulfate polyacrylamide gel electrophoresis, followed by coomassie Brilliant Blue 250 staining. Refolding was performed with cheotropic agent concentration gradient dialysis. The solution of denatured protein was dialyzed against 2 lit of freshly prepared 6, 4, 2, 1, 0 M urea with 5 mMTris (pH 7.4). With each concentration, the protein was dialyzed 12 h at 4 °C. Bradford method with bovine serum albumin (BSA) as a standard was used to assay protein concentration. Purified protein was evaluated by western blot using an anti His-tag-HRP antibody.

### Immunoreactivity of recombinant Brucella purified rVirB12 to human sera using western blotting

Recombinant protein was subjected to 12% gradient SDS-PAGE with the molecular protein marker and was transferred from the unstained polyacrylamide gel onto 0.45 µm nitrocellulose membrane. The blotted membrane blocked using 5% skim milk in TSBST 1%. After washing with TSBT, transferred proteins were immunostained with serum obtained from human brucellosis infection at a dilution of 1/1000. Secondary antibody conjugated to horseradish peroxidase was used in the assay. The reaction was visualized with enhanced chemiluminescence (ECL) and ECL system (GE Healthcare. Uppsala, Sweden).

### Production of polyclonal anti-virB12 recombinant protein in rabbit

A mature white New Zealand rabbit was immunized with purified VirB12 recombinant protein. Immunization was performed according to the protocol of Hay et al. [23]. In the first i.m injection, mixture of 250 µg recombinant protein with the same volume of Freund’s complete adjuvant was injected. For second injection the rabbit was injected with 125 µg recombinant protein with the same volume of Freund’s incomplete adjuvant, 1, 2 weeks later. Finally, 2 weeks after the last immunization, blood was collected and sera separated.

### ELISA test with commercial kit

To investigate the serological status of the samples from human and evaluate the quality of the detection method, all of 100 serum samples were subjected to ELISA kit (IBL, Germany). The plate wells of IBL ELISA kit were coated with a bacterial lysate of B.abortus strain w99 as the antigen. Ref comparsion of four commercial IgM and IgG ELISA kits for diagnosing brucellosis.

### Immunoreactivity of recombinant Brucella virB12 to human sera using ELISA

Positive samples were from the patients with a positive ELISA test. Clinical sera from human were analyzed by indirect ELISA using recombinant VirB12 as antigen. The immunoassay plates (Maxisorp, nunc, Denmark) were coated with purified recombinant VirB12 protein at a concentration of 5 µg/ml in PBS and incubated at 4 °C, overnight. The wells emptied and washed three times with phosphate buffer saline-Tween20 (PBST) and then blocked with 5% skim milk for 2 h at 37 °C. Plates were filled with sera at a dilution of 1/100 and incubated at 37 °C for 1 h. After washing with PBST for five times the plates were incubated with HRP conjugates for 1 h at 37 °C. After washing with PBST, the wells of plates were charged with substrate solution containing TMB (3,3′,5,5′-tetra methyl benzidine). Color development was stopped by adding H_2_SO_4_ 20%, after 10 min of incubation of the plates in dark at room temperature. Absorbance was measured at 450 nm wavelength in an ELISA reader. Each samples run in duplicate. Additionally, as a control for each serum, wells were left uncoated. To determine the cut off value for ELISA 60 known positive and 40 known negative sera for human brucellosis were used.

### Evaluation of ELISA method against commercial ELISA

The sensitivity, specificity, positive predictive value and negative predictive value of recombinant VirB12 ELISA for serodiagnosis of brucellosis were evaluated in comparison to commercial ELISA kit.

## Results

### Preparation and recognition of Brucella VirB12 recombinant protein

**Fig. 1:**
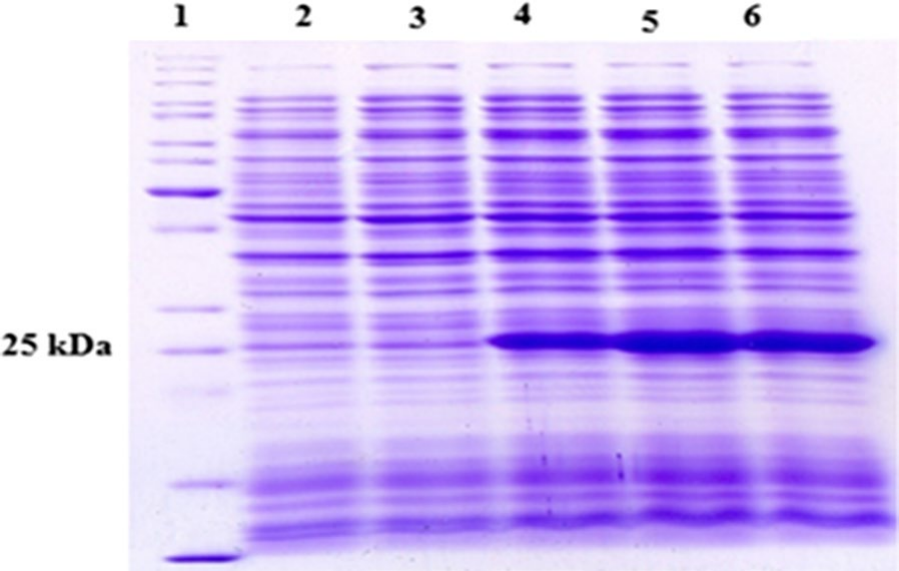
Analysis of expressed VirB12 recombinant protein at different times after induction on SDS-PAGE (12% w/v). *Lane 1* protein molecular mass marker (KDa). *Lane 2* protein expression in Bl21 (DE3) with pET28a vector 1 h after adding IPTG. *Lane 3* protein expression in transformed Bl21 (DE3) with pET28a-VirB12 recombinant vector before adding IPTG. *Lane 4* protein expression in transformed Bl21 (DE3) with pET28a-VirB12 recombinant vector 1 h after adding IPTG. *Lane 5* protein expression in transformed Bl21 (DE3) with pET28a-VirB12 recombinant vector 2 h after adding IPTG. *Lane 6* protein expression in transformed Bl21 (DE3) with pET28a-VirB12 recombinant vector 4 h after adding IPTG

**Fig. 2:**
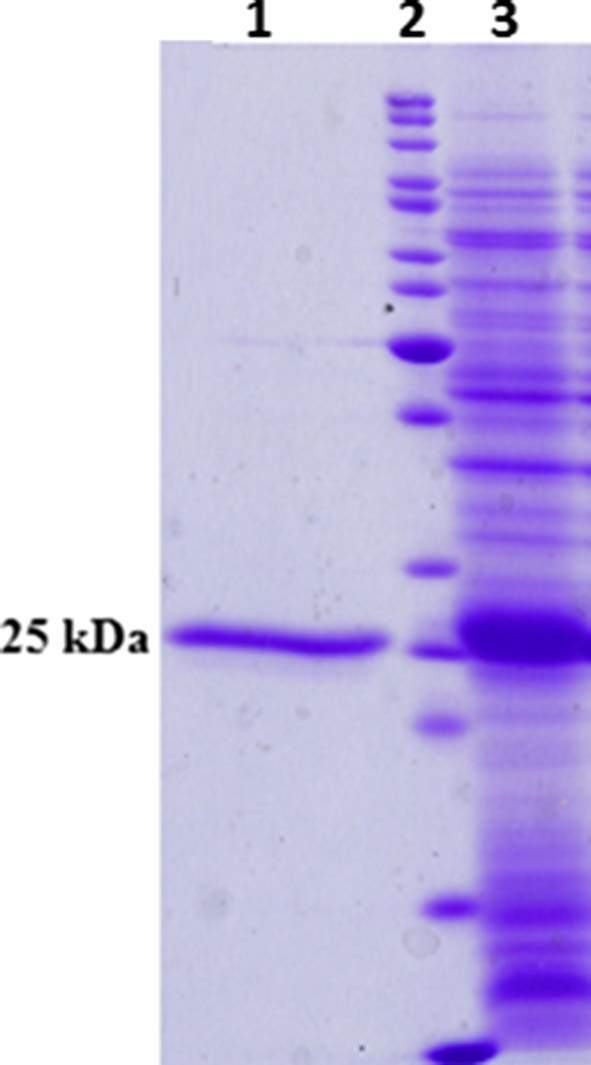
SDS-PAGE analysis of recombinant VirB12 protein expression and its purification. *Lane 1* elution of recombinant VirB12 protein through Ni-NTA column. *Lane 2* protein molecular mass marker (KDa). *Lane 3* protein protein expression in transformed Bl21 (DE3) with pET28a-VirB12 recombinant vector 3 h after adding IPTG

The pET28a-VirB12 recombinant plasmid was transformed in Bl21 (DE3) *E. coli* cells. Bacteria harboring recombinant plasmid were grown in LB medium. They were induced with 1 mM IPTG to express target recombinant protein. Samples were taken before and at 1 h intervals after induction. Total protein was electrophoresed on 12% SDS PAGE gel. SDS-PAGE analyses showed the expected molecular mass of approximately 25 kDa fusion recombinant protein. The recombinant VirB12 was mostly accumulated in the cytoplasm of *E. coli* transformant as inclusion bodies which could only by extracted and purified under denaturing condition using 8 M urea (Fig. 1). The recombinant VirB12 was mostly accumulated in the cytoplasm of *E. coli* transformant as inclusion bodies, which could only by extracted and purified under denaturing condition using 8 M urea. Following purification of rViB12 by Ni-NTA affinity chromatography, the yield of the purified protein was estimated by Bradford method to be about 0.6 mg/ml of culture (Fig. 2).

### Immunoreactivity of recombinant Brucella purified rVirB12 to human sera using western blotting

**Fig. 3:**
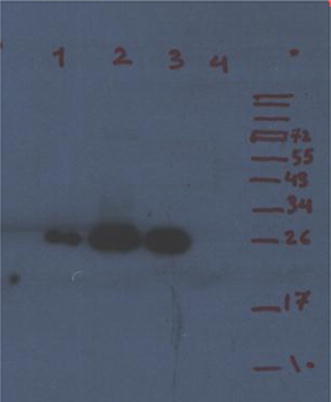
Western blot analysis against recombinant VirB12 protein by infect patient sera. *Lane 1* western blotting with patient positive Brucella serum (1/500 diluation). *Lane 2, 3* western blotting with patient positive Brucella serum (1/100 diluation). *Lane 4* western blotting with normal individual

To evaluate immune reactivity of the purified protein, western blot was performed with sera (1/1000 dilution) from *Brucella* infected human. The sera from human reacted to purified recombinant VirB12. Antigenicity of the expressed protein was confirmed by western blot analysis using patient sera. The specific antibody response from five patient sera was observed. Serum samples from normal individual was also tested as negative control and no anti VirB12 antibodies were detected. Additionally, there was no reactivity between the expressed pET28a in *E. coli* Bl21 (DE3) with patient serum (Fig. 3).

### Production of polyclonal anti-virB12 recombinant protein in rabbit

Increasing the antibody titers to high level after third boost was confirmed the good immunogenicity of VirB12. The rabbit antiserum was able to recognize the virB12 in Brucella lysate and the purified recombinant VirB12.

### Evaluation of recombinant VirB12 ELISA

Immune reactivity of recombinant virB12 was determined using an indirect ELISA. A total 100 serum samples (66 commercial-ELISA positive and 34 commercial-ELISA negative) were collected from different region of the country and were tested by indirect ELISA against the recombinant VirB12 antigen. The VirB12 ELISA was considered positive only if the mean absorbance value was greater than two SDS above the mean value for healthy serum samples. The cut of value was 0.325 (mean, 0.17 SD, 0.077). Out of 100 serum samples tested, 60 (60%) were positive and 40 (40%) were negative by ELISA. The sensitivity, specificity, accuracy, negative predictive value and positive predictive value of recombinant VirB12-based ELISA related to the commercial-ELISA method are shown in Table 1.

**Table 1:** Evaluation of diagnostic value of recombinant VirB12 antigen-based ELISA against commercial ELISA

	Commercial ELISA positive	Commercial ELISA negative	Total
ELISA-virB12 positive	58	2	60
ELISA-VirB12 negative	8	32	40
Total	66	34	100

Sensitivity: true positive/true positive + false negative*100 = 87.8%

Specificity: true negative/true negative + false positive*100 = 94%

Positive predictive value: true positive/true positive + false positive*100 = 96.6%

Negative predictive value: true negative/true Negative + False positive*100 = 80%

Accuracy: true positive + true negative/total number*100 = 90%

## Discussion

Despite using vaccination in livestocks, brucellosis remains as an endemic infection in many developing countries such as Iran. Human and animal infection with *Brucella* inflicts an enormous cost to people and government [24]. Since culture method for diagnosis of Brucellosis is time consuming and has infection risk for laboratory workers, serological tests are commonly used for clinical diagnosis [25]. Due to cross reactions with many other Gram negative bacteria in most routinely used LPS-based serological tests, identification of more specific *Brucella* spp. protective antigens can be useful for developing serological tests which avoid the drawbacks of classical ones. The outer membrane proteins in Gram negative bacteria have particular significance as a potential immunity target [26]. Brucella VirB12 is one of these structural protein which is expressed during Brucellosis infection. Investigators had demonstrated that VirB12 protein acts as an immunogen and induces partial immunity in animal models [27]. However, there is not any report concerning antigenicity of the recombinant VirB12 protein in human. VirB12 protein is located on the bacterial cell surface and is highly conserved among Brucella isolates. Therefore, VirB12 can be considered as an antigenic candidate for serological diagnosis of brucellosis. Use of recombinant VirB12 instead of extracted VirB12, is less time consuming, has a high yield and avoids handling with live pathogenic *Brucella*. In the present study, high level expression of VirB12 protein was carried out by the means of pET28a based on T7 promoter transcription translation signals in conjunction with suitable host cell *E. coli* Bl21 (DE3). The results of SDS-PAGE demonstrated that an IPTG concentration of 1 mM and 4 h of incubation under shaking condition was optimum for expression of protein. The cloning of *VirB12* gene in the pET28 system led to expression of a protein with size of approximately 25 kDa. The expressed protein contained 6 His Tag which is linked to the C-terminal of protein. These additional amino acids increase the size of expressed protein by 8 KDa. The presence of His tag sequence in the target protein also provides the possibility for purification through Ni-NTA affinity chromatography. Metal affinity chromatography was subsequently performed to purify VirB12, producing amount 3 mg of pure recombinant protein per liter of bacterial culture. Antigenicity of the purified protein component was evaluated in immunoblotting with human brucellosis sera. Data showed that the recombinant VirB12 protein could be detected as an antigenic component by sera from acute phase of human brucellosis. By using western blot analysis, we showed that the recombinant VirB12 did not shown any cross reactivity with normal human sera. There was also no serological interference related to the fused amino acids. The results, in consist of other researcher showed that there was no interference related to fused amino acids [12]. To examine the practical value of VirB12, 100 positive and negative serum samples from different part of country used in virB12 antigen based indirect ELISA for detection of antibody response against *Brucella*. The finding demonstrated that the virB12 recombinant protein had good immunogenicity and indicates that the availability of virB12 to the immune system. The Pearson correlation coefficient of the virB12 antigen-based indirect ELISA against commercial ELISA, a test based on LPS antigen was 0.73 (P < 0.001). The sensitivity of the virB12-ELISA was 87.8% and specificity was 94%. The accuracy in all samples reached 90%. The sensitivity and specificity of virB12 ELISA in comparison with commercial ELISA confirmed the fact this system comparable to the commercial assay available as a potential immunogenic marker for screening of brucellosis and needs further evaluation.

## Conclusions

In summary, our results showed that VirB12 was expressed at high amounts in *E. coli* Bl21 and could be purified by Ni-NTA affinity chromatography. This recombinant protein reacted strongly with sera from patient with brucellosis by western blot. Thus we concluded that VirB12 is antigenic and has property to be considered as a suitable candidate for development of more specific diagnostic tests.

## Abbreviations

ELISA: enzyme-linked immunosorbent assay
LPS: lipopolysaccharide
LB: Luria-Bertani
IPTG: isopropyl-β-d- thiogalactopyranoside
BSA: bovine serum albumin
ECL: enhanced chemiluminescence
PBST: phosphate buffer saline-Tween
TMB: 3,3′,5,5′-tetra methyl benzidine
